# Correlation between Traditional Chinese Medicine Syndromes and Type 2 Myocardial Infarction in Critically Ill Patients with Pulmonary Disease

**DOI:** 10.1155/2022/9329683

**Published:** 2022-03-21

**Authors:** Sheng-Li Ma, Shan-You Hu, Wu-Lin Li, Da-Li You, Ting-Ting Jiang, Li Wang, Fei Wang, Xiao Wu

**Affiliations:** ^1^Graduate School, Shanghai University of Traditional Chinese Medicine, Shanghai, China; ^2^Emergency Department, Jiading District Central Hospital Affiliated Shanghai University of Medicine & Health Sciences, Shanghai, China; ^3^Department of Critical Care Medicine, Jiading District Central Hospital Affiliated Shanghai University of Medicine & Health Sciences, Shanghai, China

## Abstract

**Background:**

Treatment based on syndrome differentiation under the traditional Chinese medicine (TCM) framework has been shown to be helpful in patients with coronary artery disease. We hypothesized that syndrome types could predict the risk of type 2 myocardial infarction (T2MI) caused by imbalance between myocardial oxygen supply and demand in critically ill patients with pulmonary disease.

**Methods:**

This retrospective study included consecutive critically ill patients with pulmonary disease admitted to the ICU at Jiading District Central Hospital Affiliated Shanghai University of Medicine & Health Sciences from January 1, 2017, to July 1, 2019. Diagnosis of T2MI was based on the fourth universal definition of myocardial infarction. Risk factors associated with T2MI were identified using multivariate regression analysis.

**Results:**

A total of 244 patients were included in the study: 78 who developed T2MI and the remaining 166 who did not develop T2MI during hospitalization. The incidence of phlegm syndrome and deficiency syndrome was 61.9% and 38.1%, respectively. In comparison with the patients with phlegm syndrome, the incidence of T2MI in patients with deficiency syndrome is significantly higher (40.9% vs. 26.5%, *P*=0.019). In multivariate logistic regression, T2MI was independently associated with the baseline troponin level (OR 12.682, 95% CI 1.397∼115.121; *P*=0.024), hemoglobin < 55 g/L (OR 12.76, 95% CI 2.359∼69.021; *P*=0.003), mechanical ventilation (OR 2.244, 95% CI 1.029∼4.892; *P*=0.042), and TCM deficiency syndrome (OR 2.214, 95% CI 1.032∼4.749; *P*=0.041). After adjusting for confounding factors in Cox regression models, the hazard ratio (95% confidence interval) of qi deficiency syndrome groups was 1.183 (95% CI 1.053∼3.123, *P*=0.032).

**Conclusions:**

Patients with deficiency syndrome are at high risk of T2MI, especially those combined with qi deficiency syndrome.

## 1. Introduction

Type 2 myocardial infarction (T2MI) refers to MI caused by an imbalance between myocardial oxygen supply and demand in the absence of occlusion of the coronary arteries [1]. Conditions that decrease oxygen supply and/or increase oxygen demand of the heart, e.g., anemia, hypotension, and arrhythmia, increase the risk of T2MI [2]. In comparison with the classic type 1 myocardial infarction (T1MI), T2MI has higher mortality [3] and worse long-term prognosis [4, 5]. In a previous study from this research group with critically ill elderly patients, the incidence of T2MI was 24.2%, and the mortality was 39% [6].

Traditional Chinese medicine (TCM) has been widely used in the treatment of critically ill patients with cardiovascular diseases [7], and traditional Chinese medicine could protect the function of zang-fu organs and treat cardiovascular diseases [8–10]. Syndrome differentiation is the cornerstone of TCM, and treatments based on syndrome differentiation have been shown to reduce the mortality and improve prognosis in patients with severe coronary heart disease [11]. In comparison with non-ST-segment elevation myocardial infarction, qi deficiency syndrome and yin deficiency syndrome are more prevalent in patients with ST-segment elevation myocardial infarction [12]. Also, MI patients with Yin deficiency tend to have more severe myocardial damage, longer hospital stay, and higher mortality rate [13]. Yin deficiency syndrome is more common in elderly population [14]. Different traditional Chinese medicine syndrome types lead to different disease characteristics and risks.

Pulmonary diseases, such as acute exacerbation of chronic obstructive pulmonary disease, acute lung injury, acute respiratory distress syndrome, sepsis from pulmonary infections, and severe pneumonia, are often seen in critically ill patients [15, 16]. There usually exists the imbalance between oxygen demanding and supplying. Meanwhile, T2MI is a common complication in critically ill patients [17]. Therefore, we speculated that the higher risk of T2MI in critically ill patients with severe pulmonary diseases could be associated with distinct TCM syndromes, and we conducted a retrospective analysis to examine the relationship between types of TCM syndrome and the risk factors of T2MI.

## 2. Materials and Methods

### 2.1. Patients

We screened all adult patients (18 years of age or older) admitted to the intensive care unit (ICU) of Jiading District Central Hospital Affiliated Shanghai University of Medicine & Health Sciences from January 1, 2017, to July 1, 2019. For inclusion in the analysis, the following criteria must be met: (1) conform to the diagnostic criteria of Western medicine disease (e.g., acute exacerbation of chronic obstructive pulmonary disease (AECOPD), chronic obstructive pulmonary disease, respiratory failure, acute respiratory distress syndrome, severe pneumonia) and (2) APACHE II (Acute Physiology and Chronic Health Evaluation II) score ≥15 points. Patients with one or more of the following conditions were excluded from the study: (1) definite diagnosis of T1MI by coronary angiography; (2) conditions that could affect troponin levels (e.g., chronic renal failure, advanced cancer, or chemotherapy); (3) minors (age <18 years old) and pregnant women; (4) TCM syndrome cannot be reasonably determined; and (5) lung cancer, pulmonary embolism, pneumothorax, and tracheal foreign body.

### 2.2. Diagnostic Criteria

Diagnosis of T2MI was based on the fourth universal definition of myocardial infarction [18]. The primary diagnostic basis was elevation and/or decrease of cardiac troponin beyond the 99th percentile upper limit of reference value (URL), and at least one of the following criteria must be met: (1) clinical evidence of ischemia; (2) new ST-T segment change or complete left bundle branch block; (3) newly emerging pathological Q waves; (4) imaging evidence for loss of active myocardium and segmental ventricular wall motility abnormality; and (5) risk factors for imbalance of myocardial oxygen supply and demand including coronary artery endothelial dysfunction, coronary artery spasm, coronary artery embolism, tachycardia or bradycardia, anemia, respiratory failure hypotension, and hypertension (with or without left ventricular hypertrophy) [19].

#### 2.2.1. The Method of Syndrome Differentiation and Classification in TCM

The clinical data of each patient were retrospectively collected within 48 hours of admission to the intensive care unit (ICU), including the clinical symptoms, signs, laboratory data, and so on. According to the Dialectical Syndrome Factor Scale in Syndrome Elements Differentiation [20], the TCM syndromes (disease location, disease nature) were determined. The integral of the same TCM syndrome elements were added. When the cumulative score exceeds the threshold of 100, the syndrome element is determined as the final TCM syndrome element. If the number of TCM syndrome elements exceeds 8, the diagnostic criterion is the cumulative score exceeding the threshold of 150. If the score of all syndromes is less than the threshold of 100, the cumulative score of 70 is taken as the diagnostic threshold [21, 22]. The TCM syndrome elements that reached the diagnostic threshold were organically linked and combined to determine the final TCM syndrome type.

TCM syndromes were divided into deficiency and phlegm syndromes. Deficiency syndromes included qi deficiency, blood deficiency, and qi-blood deficiency syndrome. Phlegm syndromes included phlegm retention and phlegm turbidity obstruction lung syndrome.

### 2.3. Data Collection

The following data were extracted from medical records: (1) sex and age; (2) comorbidities, including hypertension, diabetes, respiratory failure, chronic obstructive pulmonary disease (COPD), hemodynamic shock, atrial fibrillation, acute renal failure, hemorrhagic event, acute respiratory distress syndrome; (3) history of percutaneous coronary intervention (PCI); (4) hospital stay; (5) APACHE II score; and (6) laboratory data of leukocyte, C-reactive protein, procalcitonin, platelet, hematocrit, albumin, creatinine clearance, and troponin.

### 2.4. Statistical Analysis

Continuous variables following normal distribution are shown as mean and standard deviation and analyzed using Student's *t*-test. Continuous variables not following normal distribution are shown as median and interquartile range (IQR) and analyzed using the nonparametric Mann–Whitney *U* test. Categorical variables are shown as percentage and analyzed using the *χ*^2^ test. Multivariate logistic regression was used to identify risk factors associated with T2MI. Statistical significance was defined as *P* < 0.05. All analyses were conducted using SPSS 24.0 software.

## 3. Results

### 3.1. Overall Data Analysis

**Figure 1:**
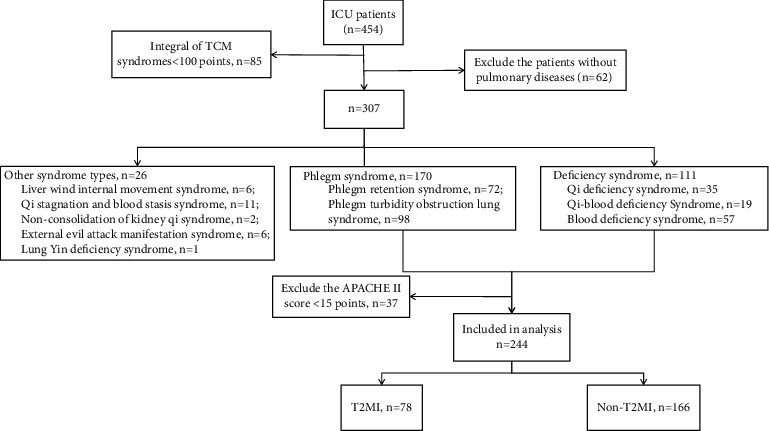
Study flowchart.

We screened a total of 454 critically ill patients, among which 210 were excluded from the final analysis for the following reasons: TCM syndrome element scores <100 points (*n* = 85), without pulmonary diseases (*n* = 62), APACHE II score <15 points (*n* = 37), liver wind internal movement syndrome (*n* = 6), qi stagnation and blood stasis syndrome (*n* = 11), nonconsolidation of kidney qi syndrome (*n* = 2), external evil attack manifestation syndrome (*n* = 6), and lung-yin deficiency syndrome (*n* = 1). The final analysis included 244 patients, 78 with vs. 166 without T2MI (Figure 1).

### 3.2. Baseline Characteristics

In the 244 patients included in the final analysis, 83 (34.0%) were men and 161 (66.0%) were women, and their mean age was 76.2 ± 13.4 years. Seventy-eight (31.9%, 78 off 244) patients had T2MI. The ICU fatality rate was 37.7% (92 of 244). Demographic and baseline characteristics of the patients are shown in Table 1. The mean APACHE II score in the patients with T2MI was higher than those with non-T2MI (24.3 ± 5.8 vs. 22.5 ± 4.8, *P*=0.012). Compared with non-T2MI patients, T2MI patients had a higher level of PCT (1.17 (0.45∼5.02) *μ*g/L vs. 0.58 (0.14∼3.58) *μ*g/L, *P*=0.014), baseline troponin level (0.140 (0.089∼0.305) ng/mL vs. 0.032 (0.017∼0.050) ng/mL, *P* < 0.001), and maximum troponin level (0.204 (0.127∼0.465) ng/mL vs. 0.036 (0.021∼0.465) ng/mL, *P* < 0.001). Meanwhile, T2MI patients had a lower estimated glomerular filtration rate (64.33 (32.33∼91.71) mL/min vs. 81.61 (51.37∼92.91) mL/min, *P*=0.008). The hospital stay of T2MI patients was longer than those with non-T2MI (12 (8∼27) days vs. 10 (6∼16) days, *P*=0.012). Furthermore, T2MI patients had a higher percentage of mortality in the ICU (56.4% vs. 28.9%, *P* < 0.001), mechanical ventilation (56.4% vs. 40.4%, *P*=0.019), and a noticeably higher rate of acute kidney injury (20.5% vs. 9.0%, *P*=0.012), compared with the patients without T2MI.

### 3.3. TCM Syndromes

The rate of phlegm syndrome and deficiency syndrome was 61.9% and 38.1%, respectively. In comparison with the patients without T2MI patients, the rate of T2MI patients with deficiency syndrome was higher (48.7% vs. 33.1%, *P*=0.019), with no significant difference in phlegm subsyndrome and deficiency subsyndrome (*P*=0.134) (Table 2).

**Figure 2:**
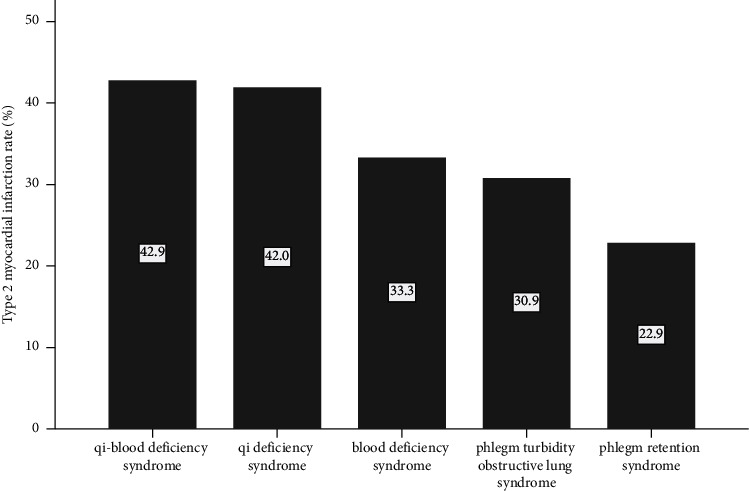
The rate of T2MI in different TCM subsyndromes.

The rate of T2MI differed significantly in patients with different TCM syndrome type: 42.9% for syndrome of deficiency of qi and blood, 42.0% for qi deficiency syndrome, 33.3% for blood deficiency syndrome, 30.9% for phlegm turbidity obstruction lung syndrome, and 22.9% for phlegm retention syndrome (*P* for trend < 0.001) (Figure 2).

In multivariate logistic regression, T2MI was independently associated with the following factors: troponine at baseline (OR 12.68, 95% CI 1.397–115.121; *P*=0.024), HB < 55 g/L (OR 12.76, 95% CI 2.359–69.021; *P*=0.003), mechanical ventilation (OR 2.24, 95% CI 1.029–4.892; *P*=0.042), and TCM syndrome of deficiency (OR 2.24, 95% CI 1.032–4.749; *P*=0.041) (Table 3).

**Figure 3:**
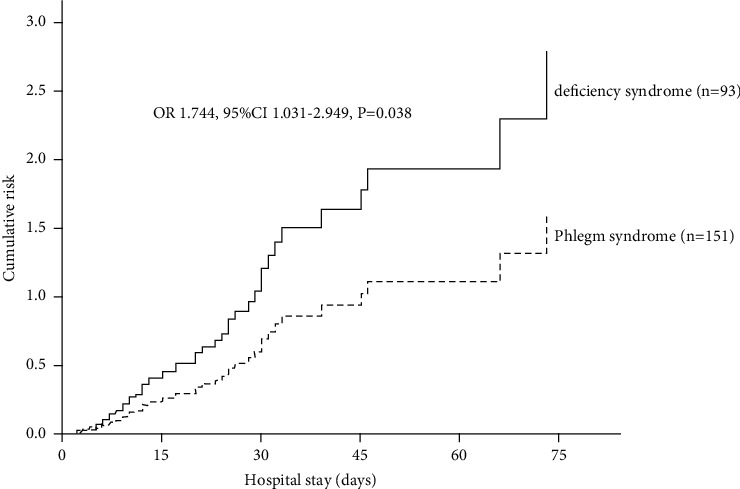
The cumulative risk of T2MI in patients with deficiency syndrome (*n* = 93) vs. phlegm syndrome (*n* = 151).

In comparison with patients with phlegm syndrome, patients with deficiency syndrome had higher cumulative risk of T2MI (OR 1.744, 95% CI 1.031–2.949) (Figure 3).

**Figure 4:**
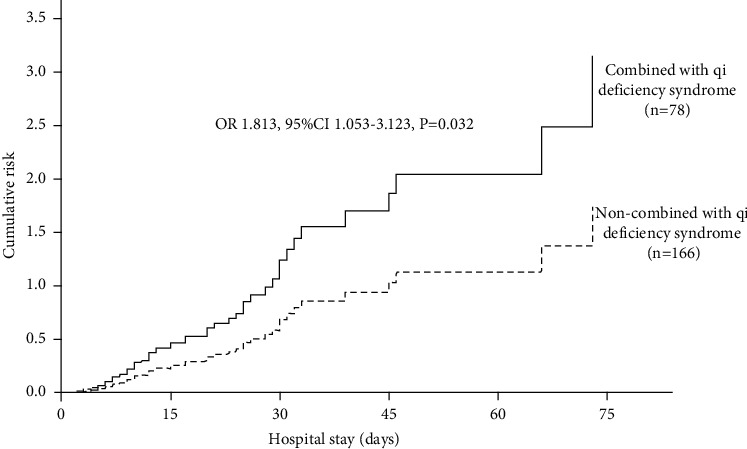
Cox regression analysis for risk of T2MI in patients with vs. without qi deficiency syndrome.

In comparison with patients without qi deficiency syndrome, patients with qi deficiency syndrome had higher cumulative risk of T2MI (OR 1.813, 95% CI 1.053–3.123; *P*=0.032) (Figure 4).

## 4. Discussion

To our knowledge, this is the first clinical research on the relationship between TCM syndrome and T2MI in critically ill patients. The major findings of our study were that the main TCM syndromes were phlegm and deficiency syndromes in critically ill patients with pulmonary diseases and there was a significantly higher risk of occurrence of T2MI in patients with deficiency syndrome, especially in those with qi deficiency syndrome.

According to “Diagnosis and Treatment Guidelines of Common Diseases in Internal Medicine of Traditional Chinese Medicine,” which was formulated in 2008 by the China Association of Chinese Medicine (CACM) [23], pulmonary diseases include “cough,” “asthma disease,” “wheezing disease,” “lung impotent,” “pulmonary abscess,” “atrophic lung disease,” and so on. The TCM syndromes classification and their diagnosis criteria were also determined. However, all the above standards are mainly based on personal experiences, which are highly subjective and lacks objective TCM syndrome differentiation indexes [24]. The TCM syndrome factor differentiation method, which determines the location and nature of the diseases through the identification of syndromes factors (symptoms and signs and other clinical information), was proposed. This method is less subjective and more practical [25] and is gradually applied to the study of TCM syndromes [26]. Meanwhile, TCM syndrome type was difficult to evaluate and determine in critically ill patients because of the complex conditions, and there are no recommendations clearly on the identification of TCM syndromes in critically ill patients. Thus, in this study, the TCM syndrome factor differentiation method was adopted to identify the TCM syndromes of critically ill patients, and it is shown that the proportion of phlegm and deficiency symptoms reached 90%, which further validated the findings of Xu's research [27].

TCM research projects have found that intermingled phlegm-stasis blood syndrome is prominent in coronary heart disease patients [28], while qi deficiency and blood stasis syndrome is the major syndrome in myocardial infarction patients [29, 30]. Treatment with *Arnebia euchroma* and *Lycium barbarum* that invigorate qi and promote blood circulation can reduce the risk of developing cardiac arrhythmia, heart failure, and thrombose in myocardial infarction patients through supplement of middle qi, antioxidation, and anti-inflammation [31–33]. Meanwhile, the incidence of heart failure after MI was decreased with the use of reperfusion therapy [33]. Beta-arrestins show benefit to treat heart failure [34]. T2MI is myocardial necrosis caused by an imbalance in myocardial oxygen supply and demand and is not associated with coronary atherosclerotic thrombosis [35]. One of the main risk factors for T2MI is hypoxemia, and the reduced partial pressure of oxygen in tissue fluid leads to metabolic imbalance, which is closely related to the occurrence of qi deficiency [36, 37]. More than 40% of patients with qi deficiency have a significant decrease in hemoglobin [38], and severe anemia is an important risk factor for the development of T2MI [39]. This may explain one of the conclusions of this study: patients with deficiency have a significantly higher risk of developing T2MI.

Further analysis showed that, compared with patients with other syndromes, patients with deficiency syndrome (OR 1.744), especially those with qi deficiency syndrome (OR 1.831), had a significantly higher risk of developing type 2 myocardial infarction. This finding strongly implied that TCM syndrome type deficiency, especially qi deficiency syndrome, has important value in predicting the occurrence of type 2 myocardial infarction in patients with critical pulmonary diseases. This suggests that close attention should be paid to deficiency syndrome, especially the occurrence of T2MI in patients with qi deficiency syndrome, in the clinical classification of TCM syndromes in critically ill patients with pulmonary diseases. At the same time, in this study, we also found that the incidence rate of acute kidney injury and length of hospital stay in patients with type 2 myocardial infarction requiring mechanical ventilation were significantly higher, which was consistent with previous studies [40, 41]; therefore, clinical attention should be paid to patients with deficiency, especially those with critical pulmonary diseases combined with qi deficiency syndrome.

The present study had several limitations: (1) although the application of TCM syndrome element differentiation method avoids the result bias caused by subjectivity, it is difficult to collect all the factors required by TCM syndrome type due to the condition characteristics of critically ill patients; (2) relatively small number of patients from a single center and the retrospective study are also important limitations; and (3) only 7.8% of the patients in this study received coronary angiography.

In conclusion, a more objective TCM syndrome differentiation method was proposed. We found that phlegm syndrome was more common than deficiency syndrome in patients with critical pulmonary diseases, while patients with deficiency syndrome, especially qi deficiency syndrome the poorest group, had a higher risk of type 2 myocardial infarction. TCM syndrome type was closely related to the occurrence of type 2 myocardial infarction in patients with critical pulmonary diseases.

## Figures and Tables

**Table 1:** Demographic and general characteristics.

Variables	All *n* = 244	T2MI *n* = 78	Non-T2MI *n* = 166	*P*
Demographic data				
Male [*n* (%)]	83 (34.0)	27 (34.6)	56 (33.7)	0.892
Age [Mean ± SD, years]	76.2 ± 13.4	76.0 ± 14.6	76.3 ± 12.9	0.851
Comorbidities [*n* (%)]				
Hypertension [*n* (%)]	137 (56.1)	45 (57.7)	92 (55.4)	0.739
Respiratory failure [*n* (%)]	118 (48.4)	43 (55.1)	75 (45.2)	0.147
Diabetes [*n* (%)]	49 (20.1)	18 (23.1)	31 (18.7)	0.423
COPD [*n* (%)]	38 (15.6)	10 (12.8)	28 (16.9)	0.416
Various types of shock [*n* (%)]	36 (14.8)	14 (17.9)	22 (13.3)	0.335
Atrial fibrillation [*n* (%)]	33 (13.5)	11 (14.1)	22 (13.3)	0.856
AKI [*n* (%)]	31 (12.7)	16 (20.5)	15 (9.0)	0.012
Hemorrhagic event [*n* (%)]	18 (9.1)	8 (10.3)	10 (6.0)	0.238
MODS [*n* (%)]	7 (2.9)	4 (5.1)	3 (1.8)	0.147
ARDS [*n* (%)]	5 (2.0)	2 (2.6)	3 (1.8)	0.697
Tachycardia (non-AF) [*n* (%)]	4 (1.6)	2 (2.6)	2 (1.2)	0.436
History of PCI [*n* (%)]	4 (1.6)	3 (3.8)	1 (0.6)	0.063
Hospital stay [M (Q1, Q3) days]	11 (6∼18)	12 (7∼26)	10 (6∼16)	0.012
Laboratory parameters				
WBC [M (Q1, Q3) × 10^9/L]	10.55 (7.75∼14.62)	11.1 (8.22∼14.9)	10.5 (7.5∼14.5)	0.245
PLT [M (Q1, Q3) × 10^9/L]	157 (107.0∼219.0)	143.5 (88.0∼217.2)	164 (113.5∼221.0)	0.07
CRP [M (Q1, Q3), mg/L]	52.12 (18.86∼121.85)	49.07 (17.55∼123.60)	53.4 (20.90∼126.00)	0.91
PCT [M (Q1, Q3), *µ*g/L]	0.719 (0.18∼4.20)	1.17 (0.45∼5.02)	0.58 (0.14∼3.58)	0.014
HCT [mean ± SD, %]	32.75 ± 7.23	32.04 ± 6.81	33.09 ± 7.41	0.302
HB < 55 g/L [*n* (%)]	12 (4.9)	10 (12.8)	2 (1.2)	<0.001
ALB [mean ± SD, g/dL]	29.83 ± 5.79	29.50 ± 5.43	29.98 ± 5.97	0.556
eGFR[M (Q1, Q3), mL/min]	77.56 (44.71∼92.79)	64.33 (32.33∼91.71)	81.61 (51.37∼92.91)	0.008
Troponin [M (Q1, Q3), ng/mL]				
Baseline	0.044 (0.021∼0.104)	0.14 (0.089∼0.305)	0.032 (0.017∼0.050)	<0.001
Maximum	0.058 (0.029∼0.145)	0.204 (0.127∼0.465)	0.036 (0.021∼0.057)	<0.001
Mechanically ventilation [*n* (%)]	111 (45.5)	44 (56.4)	67 (40.4)	0.019
APACHE II score [mean ± SD]	23.1 ± 5.2	24.3 ± 5.8	22.5 ± 4.8	0.012
Death in the ICU [*n* (%)]	92 (37.7)	44 (56.4)	48 (28.9)	<0.001

Abbreviation: COPD, chronic obstructive pulmonary disease; AKI, acute kidney injury; MODS, multiple organ dysfunction syndrome; ARDS, acute respiratory distress syndrome; non-AF, non-atrial fibrillation; PCI, percutaneous transluminal coronary intervention; WBC, white blood cell count; PLT, platelet count; CRP, C-reactive protein; PCT, procalcitonin; HCT, red blood cell specific volume; HB, hemoglobin; ALB, albumin; eGFR, estimated glomerular filtration rate; APACHE II, Acute Physiology and Chronic Health Evaluation II; ICU, intensive care unit.

**Table 2:** TCM syndromes in patients with vs. without T2MI.

	All *n* = 244	T2MI *n* = 78	Non-T2MI *n* = 166	Statistics	*P*
TCM syndrome [*n* (%)]					
Phlegm syndrome	151 (61.9)	40 (51.3)	111 (66.9)	*x* ^2^ = 5.465	0.019
Deficiency syndrome	93 (38.1)	38 (48.7)	55 (33.1)		
TCM syndrome subtype [*n* (%)]					
Phlegm retention syndrome	83 (34.0)	19 (24.4)	64 (38.6)	*x* ^2^ = 7.034	0.134
Phlegm turbidity obstruction lung syndrome	68 (27.9)	21 (26.9)	47 (28.3)		
Qi deficiency syndrome	50 (20.5)	21 (26.9)	29 (17.5)		
Qi-blood deficiency syndrome	28 (11.5)	12 (15.4)	16 (9.6)		
Blood deficiency syndrome	15 (6.1)	5 (6.4)	10 (6.0)		

Abbreviation: TCM, traditional Chinese medicine; T2MI, type 2 myocardial infarction.

**Table 3:** Multivariate logistic regression of the risks of T2MI.

Variables	OR	95% CI	*P*
Age (year)	0.989	0.959∼1.020	0.478
cTn baseline (ng/mL)	12.682	1.397∼115.121	0.024
APACHE II score	1.032	0.962∼1.107	0.385
eGFR (mL/min)	0.992	0.979∼1.005	0.213
Female (case %)	1.13	0.510∼2.506	0.763
Deficiency syndrome (case %)	2.214	1.032∼4.749	0.041
AKI (case %)	1.966	0.725∼5.334	0.184
HB < 55 g/L	12.76	2.359∼69.021	0.003
Mechanically ventilation (case %)	2.244	1.029∼4.892	0.042
History of PCI (case %)	4.533	0.365∼56.306	0.24

## Data Availability

The data sets used and/or analyzed during the current study are available from the corresponding author on reasonable request.

## Abbreviations

TCM: Traditional Chinese medicine
T2MI: Type 2 myocardial infarction
T1MI: Type 1 myocardial infarction
ICU: Intensive care unit
COPD: Chronic obstructive pulmonary disease
AECOPD: Acute exacerbation of chronic obstructive pulmonary disease
cTn: Baseline: cardiac troponin baseline
AKI: Acute kidney injury
MODS: Multiple organ dysfunction syndrome
ARDS: Acute respiratory distress syndrome
Non-AF: Non-atrial fibrillation
PCI: Percutaneous transluminal coronary intervention
WBC: White blood cell count
PLT: Platelet count
CRP: C-reactive protein
PCT: Procalcitonin
HCT: Red blood cell specific volume
HB: Hemoglobin
ALB: Albumin;
eGFR: Estimated glomerular filtration rate
APACHE II: Acute Physiology and Chronic Health Evaluation II.
